# Dopamine and the Development of Executive Dysfunction in Autism Spectrum Disorders

**DOI:** 10.1371/journal.pone.0121605

**Published:** 2015-03-26

**Authors:** Trenton Kriete, David C. Noelle

**Affiliations:** 1 Department of Psychology & Neuroscience, University of Colorado Boulder, Boulder, CO, USA; 2 Cognitive & Information Sciences, University of California, Merced, Merced, CA, USA; Heidelberg University, GERMANY

## Abstract

Persons with autism regularly exhibit executive dysfunction (ED), including problems with deliberate goal-directed behavior, planning, and flexible responding in changing environments. Indeed, this array of deficits is sufficiently prominent to have prompted a theory that executive dysfunction is at the heart of these disorders. A more detailed examination of these behaviors reveals, however, that some aspects of executive function remain developmentaly appropriate. In particular, while people with autism often have difficulty with tasks requiring cognitive flexibility, their fundamental cognitive control capabilities, such as those involved in inhibiting an inappropriate but relatively automatic response, show no significant impairment on many tasks. In this article, an existing computational model of the prefrontal cortex and its role in executive control is shown to explain this dichotomous pattern of behavior by positing abnormalities in the dopamine-based modulation of frontal systems in individuals with autism. This model offers excellent qualitative and quantitative fits to performance on standard tests of cognitive control and cognitive flexibility in this clinical population. By simulating the development of the prefrontal cortex, the computational model also offers a potential explanation for an observed lack of executive dysfunction early in life.

## Introduction

People with autism spectrum disorders (ASD) are often impaired across a range of cognitive tasks, including planning [1,2], flexibly adapting behavior [2,3], and tasks requiring spontaneous generation of novel behaviors and ideas [4]. All of these tasks have been associated with executive control processes, so the observed impairments have led some researchers to view executive dysfunction as a central feature of autism. Indeed, the Executive Dysfunction (ED) theory of autism seeks to explain many of the behavioral patterns exhibited by these individuals in terms of a failure of executive control over behavior [5].

A wealth of data provides strong support for the prefrontal cortex (PFC) being a major contributing area in executive functioning [6–9]. Along with the central claim of ED theory, this suggests that the root cause of many autistic behavioral patterns may lie in abnormalities in this region of the brain. This is an interesting hypothesis because, while substantial progress has been made in many areas of autism research, no consensus has been reached concerning the neural basis of the disorder. This view of ED suggests that the irregular development of prefrontal cortex may underly some important patterns of cognitive performance seen in autism.

However, a more detailed examination reveals that not all forms of executive processing are commonly impaired [3,10–13]. Indeed, a somewhat perplexing aspect of the executive profile demonstrated by people with autism is that cognitive flexibility has been shown to be impaired while fundamental cognitive control remains robust and relatively unaffected. Cognitive control describes our ability to enact a behavior in the presence of a distracting or more automatic competing response. In contrast, cognitive flexibility can be described as our ability to fluently adjust cognitive control as contingencies change. A classic measure of cognitive control is the Stroop task [14], and a common measure of cognitive flexibility is performance on the Wisconsin Card Sort Test (WCST) [15]. Persons with autism have been shown to exhibit poor WCST performance, but they exhibit no more interference on the Stroop task than healthy controls [2]. In fact, the distinct pattern of reduced cognitive flexibility but relatively retained cognitive control found in people with autism is very different than many patterns of executive dysfunction exhibited in other disorders. This suggests that the neural causes of executive dysfunction in autism might be substantially different than those giving rise to executive control problems in other disorders. For instance, in schizophrenia, control is impaired in tasks such as Stroop. Some have attributed this deficit to an inability to actively maintain the proper contextual information in PFC, resulting in a lack of critical top-down guidance needed to overcome more prepotent processing pathways [16,17]. In patients with frontal damage, both control and flexibility are impaired, in comparison to controls [7,8]. Yet another pattern of results is seen in Attention Deficit Hyperactivity Disorder (ADHD), where deficits have been observed in inhibitory control without significant deficits in cognitive flexibility [2]. These varied observations suggest that executive processes include multiple components that can be selectively damaged. Specifically, the appearance of impairments in cognitive flexibility combined with spared cognitive control in autism challenges the most basic conjecture that autism involves a global dysfunction of executive processes, perhaps due to frontal abnormalities.

A second challenge appears in the developmental trajectory of executive deficits in autism. In young children with autism, executive abilities appear developmentally appropriate when compared with controls matched for age and verbal ability [18], calling into question the role of ED in the etiology of autism. Any theory intending to explain executive dysfunction in autism must account for the relative cognitive “strengths” and “weaknesses” that have been observed, as well as for the lack of observable deficits early in development.

One clear approach to explaining the executive profile seen in ASD involves positing separate mechanisms for cognitive control and for the flexible adaptation of control. In autism, the mechanism for control might be intact, but the mechanism responsible for the flexible adjustment of control might be compromised. Interestingly, this segregation of function is captured by an existing computational model of the prefrontal cortex and its role in executive processing: the *Cross-Task Generalization model (XT)*. Driven by broad neurocomputational considerations, XT casts the prefrontal cortex as central to cognitive control, while interactions between the PFC and the mesolimbic dopamine (DA) system mediate the flexible adaptation of control. The XT model has previously been used to capture the performance of both frontally damaged individuals and healthy controls on both the Stroop task and WCST [19].

In this report, we demonstrate how the separation of function and mechanisms that is elucidated in the XT model can help explain the executive processing profile exhibited by persons with autism. Specifically, we hypothesize that the unique patterns of executive performance in people with autism can be explained by considering the detailed mechanistic implementation of control and flexibility separately, in terms of active maintenance of representations in the PFC for control, and the updating of these representations within the PFC for flexibility. This is in contrast to the more general executive dysfunction theory, where executive control processes are not defined precisely. To investigate our hypothesis, we demonstrate that weakening the influence of DA on pyramidal cells in PFC in the XT model is sufficient to both qualitatively and quantitatively capture autistic performance on both Stroop and WCST. This suggests that the specific executive profile demonstrated by people with autism may be mediated by PFC/DA interactions. Importantly, XT is a learning model, in which the development of neural representations and associated behavioral performance can be tracked as the model matures. Leveraging this property of XT, we show that the late appearance of executive deficits might be explained by the late maturation of PFC representations and PFC/DA interactions. According to the model, early performance is driven largely by non-frontal, more posterior, brain systems which are largely unaffected by the posited DA-related abnormalities in autism. As PFC becomes more effective, differences in PFC/DA interactions are unmasked.

## Background

### Gating the Prefrontal Cortex

PFC has been broadly implicated both in cognitive control and cognitive flexibility [7,8]. Under some accounts, cognitive control depends upon the active maintenance of abstract rule-like representations in PFC. These sustained PFC representations provide a top-down task-appropriate processing bias to more posterior brain areas [9,20]. Behaviorally, this is believed to provide an avenue to guide our behavior in a manner consistent with the maintained representation within the PFC. Biologically, the active maintenance of frontal control representations is supported by dense patterns of recurrent excitation in the PFC, as well as intrinsic maintenance currents [6,21–23]. Analyses of these neural circuits have shown that active maintenance and the flexible adaptation of control are at odds. The mechanisms that maintain PFC representations, and protect them from distracting inputs, act as an obstacle to the rapid updating of PFC contents in response to shifting contingencies. In other words, the ability to focus on a goal or specific aspect of a task is at odds with the ability to switch the goal as our environment changes. Thus, in order to achieve flexible behavior, a separate process is needed to intelligently and rapidly update the actively maintained PFC control representations in a task appropriate manner. This process has been described as an *adaptive gating mechanism*. This mechanism learns, from experience, when PFC contents should be protected from interference, effectively closing the “gate” between PFC and its inputs, and when PFC contents should be rapidly updated, opening the “gate” to allow inputs to change PFC neural firing patterns and subsequently change the focus that is guiding our behavior to some other relevant rule or goal [24]. The XT model includes such an adaptive gating mechanism, with the process of learning when to update PFC contents mediated by the midbrain dopamine system [19]. We hypothesize that the effect of this gating mechanism on PFC is central to understanding executive dysfunction in autism. The adaptive gating mechanism is described next.

### Dopamine & Temporal Difference Learning

The firing of mesolimbic dopamine (DA) neurons has long been associated with reward. Evidence continues to accumulate for a more nuanced interpretation of these cells, however, in which DA encodes a measure of *change in expected future reward* [25,26]. This interpretation is important because it links biological observations to the machine learning literature, where a measure of change in expected future reward is called the *temporal difference (TD) error*. The TD error plays a central role in a class of reinforcement learning algorithms known as *temporal difference (TD) learning* [27]. This connection has led researchers to formalize the role of midbrain DA neurons in learning from rewards [25,28], equating the firing rate of DA cells with the TD error, defined as:

$$\begin{array}{c} \delta (t)\;=\;r(t)\;+\;\gamma \;V(t+1)\;-\;V(t) \end{array}$$ (1)

…where *r*(*t*) is a measure of the reward delivered to the organism at time *t*, *V*(*t*) and *V*(*t* + 1) are measures of expected future reward (“value”) for two consecutive time steps as estimated by an *adaptive critic (AC)* neural circuit (perhaps instantiated largely in orbitofrontal cortex), and *γ* is a *discount factor*, between zero and one, which controls the degree to which a long wait for a reward reduces its effective value to the organism. Note that the TD error is defined so that, if the AC predicts future reward perfectly, the value of *δ*(*t*) will consistently be zero. A positive TD error indicates that the organism is receiving, or is now expecting, more reward than it was expecting during the previous time step. Similarly, a negative TD error indicates that the organism is receiving, or is now expecting, less reward than it was expecting during the previous time step. Using TD learning, the value of *δ*(*t*) can be used to drive learning in the AC, improving estimates of *V*(*t*) with experience. The same TD error can also be used to drive learning in other adaptive neural networks which select actions, improving the organism’s selection of actions so as to lead to larger expected rewards [28]. In addition to acting as a powerful neural learning signal, influencing synaptic plasticity, the TD error also acts as a good cue for updating PFC contents [24]. In short, increased firing of the mid-brain cells indicate that things are going better than expected, and the control representations maintained within the PFC should be strengthened (positive TD Error). When things are going worse than expected (negative TD Error), then it is time to release the contents being maintained within the PFC and to try something else. In the next section we describe how this biological system is implemented within the XT framework.

### The XT Model

**Fig 1:**
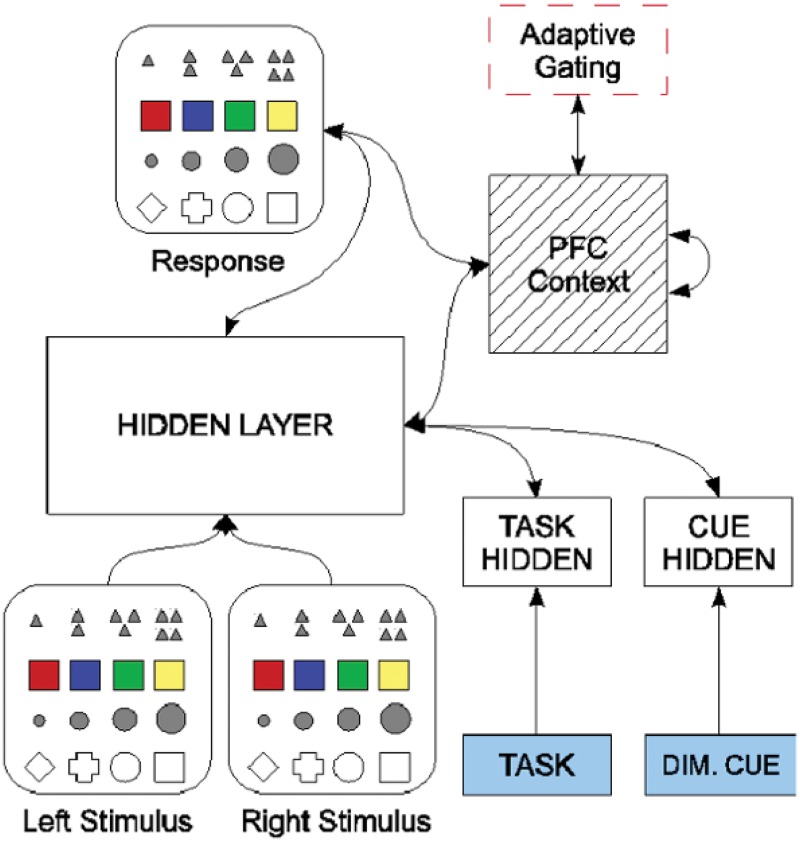
The XT Model Architecture. Boxes represent layers of simulated neural processing units. Arrows represent complete connectivity from all of the neurons in one layer to all of the neurons in another. Fast, pooled, lateral inhibition is implemented within each layer, but is not shown. Note the recurrent excitatory connections present in the PFC layer.

The architecture of the XT model is shown in Fig 1. This computational cognitive neuroscience model makes use of the biologically grounded Leabra framework [29]. Leabra is a biologically realistic, computational cognitive neuroscience modeling framework. The software that implements this framework is freely available for public use and is actively supported [30]. The input of XT consists of two layers of neural units that can be used to specify the presentation of up to two stimulus objects. It is natural to think of the rows of each input layer as representing different stimulus dimensions (e.g., color, shape, texture) and the columns indexing features across each dimension (e.g., red, orange, green, blue). The output Response layer has essentially the same structure as an input layer, with strong lateral inhibition among response units allowing the network to output a single stimulus feature (e.g., red) at a time. (The tasks to be performed by this network are described below.)

A collection of additional processing units called “Hidden” layers are used to model various posterior cortical circuits that map from stimuli to responses. Importantly, information processing in these layers can be adjusted using top-down signals from an actively maintained representation in the PFC layer. Unlike previous computational models, the representations that can be maintained in PFC are learned through a “development” process that involves extensive training on a variety of simple tasks. These developmental tasks all share a need to selectively attend to individual dimensions of the stimuli. For instance, the network might be presented with a stimulus pattern representing a large red circle and a small red square. Through input activity at the Task layer, the network might be asked to identify the feature that is the “same” across these two stimuli. In this case, the network must focus upon the “color” dimension of the stimuli and respond by activating the “red” unit in the Response layer. On each trial, corrective feedback is provided to adjust the sensitivity of modeled neurons to their inputs, driving the synaptic learning processes. In addition to this supervised learning process, correct responses from the network result in an external reward signal (*r*(*t*)), that is used by the adaptive gating mechanism described below. Over time, this developmental training process causes the network to selectively activate the hidden layer representations of relevant stimulus dimensions, allowing the network to effectively attend to a single dimension at a time [19].

The Task input to the network is used to indicate which task is to be performed, with one input unit coding for each task. Tasks included operations like *naming* a feature (e.g., “What color is this stimulus?”), *matching* two stimuli (e.g., “Are these two stimuli of the same shape?”), and *comparison* of features (e.g., “What is the size of the larger of these two stimuli?”). The Dimension Cue layer is used to inform the network of the currently relevant stimulus dimension. For example, the Dimension Cue input is used to model the Stroop task by informing the network when it should attend to the color of the stimulus rather than its word form, or vice versa. Each unit in the Dimension Cue layer corresponds to a stimulus dimension, and all of these inputs are turned off for tasks in which the network must discover the relevant stimulus dimension on its own, such as in WCST. In the original XT model, each stimulus has five dimensions, with four feature levels per dimension, and four different tasks are used during developmental training of the model.

The flexible adjustment of cognitive control is implemented using a DA-based adaptive gating (AG) mechanism. The special AG unit receives its input from the PFC layer and calculates, using learned synaptic strengths from the PFC to the AG, to produce a *V*(*t*) value — an estimate of the amount of reward the network should expect given the current PFC state and past experience. The synaptic weights on connections leading into the AG are adjusted based on the TD error, *δ*(*t*), using a neural implementation of the TD learning algorithm [19]. Importantly, the TD error, which encodes a DA signal, is also used to determine when PFC contents are to be updated. This adaptive gating process is performed in the following manner.

When the model receives more reward than expected, *δ*(*t*) is greater than zero. This results in the current PFC representation being strengthened using an intrinsic maintenance current to stabilize the existing pattern of activity. The actively maintained control state can subsequently affect processing in a manner favoring actions consistent with the contents of PFC (e.g., shift attention in the Hidden layers to a relevant stimulus dimension). When the model performs worse than expected (*δ*(*t*) < 0), the current PFC representation is destabilized, allowing a new, possibly more appropriate PFC representation, based on the inputs into the PFC layer, to be entertained. Over time, the network learns to maintain PFC representations that are likely to result in reward.

The XT model is the first computational cognitive neuroscience model to explore the development of PFC representations, and it is the first to provide good quantitative fits to both Stroop and WCST data in a single model, for both neurologically intact and frontally damaged people, based on a biologically informed architecture. Utilizing the XT modeling framework, we have investigated if reducing the overall ability of DA to stabilize and destabilize the representations contained within the PFC is sufficient to explain the specific patterns of executive dysfunction demonstrated by people with autism.

### Dopamine Abnormalities

The evidence for neurotransmitter abnormalities in ASD is varied, but, taken as a whole, worth further consideration. Abnormal DA levels have been demonstrated through many measures including PET, measures of DA metabolites such as homovanillic acid (HVA), and through the use of DA modulators in clinical trials demonstrating some mitigation of problematic behavior in ASD [31–33]. Recently, studies have found potential genetic differences in specific dopamine receptors as well as evidence that differences in the morphology of the basal ganglia is potentially relevant in ASD [34,35]. While the precise causal role, if any, that DA plays in autism is still an active area of investigation, this neurotransmitter does have interesting ties to numerous core deficits and related symptoms in autism spectrum disorders, including seizures [36,37], motor problems [38,39], repetitive behaviors [40–42], and neurogenesis [43]. This broad range of DA-related symptoms that are often observed in ASD suggests a potentially larger role for DA in ASD than has been previously considered.

### WCST

The WCST makes use of a deck of cards, where each card is imprinted with a stimulus that can vary along three dimensions (e.g., color, shape, and quantity) and across four different feature levels per dimension (e.g., for the color dimension: red, orange, green, & blue). Participants are told to sort the cards into piles, but they are not given any instructions concerning how to do this correctly. Instead, only sparse performance feedback — “Correct” sort or “Incorrect” sort — is given upon the placement of each card, with feedback based on a sorting criterion (i.e., one of the stimulus dimensions) known only to the experimenter. This process continues until the participant discovers the hidden “correct” sorting strategy. After this sorting rule (e.g., sort by color) is learned by the participant, as demonstrated by 10 consecutive cards sorted correctly, the sorting rule is changed without informing the learner. This procedure continues until either 6 correct categories (sets of 10 correct consecutive sorts) are achieved, or all 128 cards in the deck are exhausted. Errors are recorded as incorrect sorts, with *perseverative errors* scored as incorrect sorts that would be considered correct according to the previous sorting rule. Success at WCST requires the ability to flexibly change the stimulus dimension of interest. From the perspective of the XT model, this ability involves updating the sorting rule actively maintained in PFC as contingencies change.

### Stroop

The Stroop task tests cognitive control by measuring the ability to inhibit a prepotent response. In Stroop, the stimuli are different words presented in various colored fonts. Participants are asked to either read the word or to name the color of the font in which the text is presented. People are faster overall at reading the word as opposed to naming the color of the word. Furthermore, when comparing a neutral condition (e.g., the word “house” in red font) with an incongruent condition (e.g., the word “green” written in red font), people are slower in the incongruent case for color naming, but not for word reading. The magnitude of this increase in response time is known as *Stroop interference*.

[16] provided a seminal computational account of human performance on the Stroop task. Their model incorporated separate word reading and color naming pathways through posterior cortical areas, with the word reading pathway made more automatic by strengthening the synaptic strengths along that pathway. A task representation — either “word reading” or “color naming” — was actively maintained in a layer of simulated PFC neurons, providing top-down activity into the pathway corresponding to the current task. When performing the “color naming” task, this top-down activity was necessary to consistently overcome the stronger word reading pathway, and, in the incongruent condition, response competition between the inherently stronger pathway (word reading) and the pathway supported by top-down PFC activity (color naming) resulted in an increase in the time needed to activate an output response. Response time was not elevated in the word reading incongruent condition, however, as the weaker color naming pathway, when left without activation support from PFC, offered little competition for the stronger word reading pathway.

## Methods

There were no human participants in our study. This paper is a computational model of brain function of people with autism. Therefore, no IRB approval was required. A total of 100 XT model networks were prepared using the standard XT developmental training procedure, stopping when the network achieved a generalization performance criterion of no more than 10% error on a random sample of 250 trials involving novel stimulus items, or after a maximum of 100 training epochs [19], where training epoch involved training for 2000 trials. Two versions of each network were then examined: one with normal DA modulation (*κ* = 1.00), and one with reduced DA modulation (*κ* = 0.54). Each resulting network was treated as an individual experimental participant for the purposes of data analysis. This resulted in a sample size of (n = 100) for the control group and (n = 100) for the group simulating performance of people with autism. Each “individual” was tested on both the WCST and the Stroop task.

### Gating Mechanism

Based on the conceptual framework provided by the XT model, we hypothesize that a deficit in DA functioning can account for the impaired cognitive flexibility seen in people with autism, while leaving cognitive control robust and relatively unaffected. We have tested the plausibility of this conjecture by reducing the effect of the DA signal in the XT model. Specifically, we scale the neural signal provided by the DA system (TD error) by a constant factor, *κ*, where *κ* = 1 for models of healthy individuals and *κ* < 1 for models of people with autism.

$$\begin{array}{c} \delta (t)\;=\kappa (\;r(t)\;+\;\gamma \;V(t+1)\;-\;V(t)) \end{array}$$ (2)

The scaling of *δ*(*t*) by *κ* is the *only* modification from the original XT model that we have made in these simulations, introducing only a single free parameter. A parameter search revealed a *κ* value of 0.54 produced the best fit to human performance, so this value was used in all of our simulations. The paramter fitting consisted of a simple grid search, between .5 and 1.0 adjusting *κ* by intervals of.1 between these values. Below a value of *κ* =.5 the network failed to reach criterion during training. Between the values of.5 and 1, the networks demonstrated a monotonic increase in perseverative errors as described in the WCST section below. This reduction in the strength of the DA signal can be seen as decreasing the efficacy of the PFC adaptive gating mechanism, resulting in the less efficient destabilization of PFC when errors are unexpectedly made.

### Modeling WCST

Modeling WCST using the XT model required use of only three of the five possible input stimulus dimensions, which can be thought of as representing dimensions such as color, shape, and numerocity. The protocol for administrating WCST to human participants was followed without modification in these simulations. The network was presented with one stimulus item at a time, representing the current card to be sorted. The task of the network was to name the currently relevant feature (e.g., the feature “red” if color is the currently relevant sorting dimension and the current stimulus is red). No explicit information was provided to the network concerning the current correct sorting dimension, leaving the network to use a more-or-less random search strategy until the correct rule was discovered. The only information about the correct rule that was available to the network took the form of sparse feedback — “reward” or “no reward” — with reward (*r*(*t*) = 1) only arriving upon each correct sort. The XT model is able to leverage this feedback through its DA based AG mechanism, coupled with the active maintenance and top-down modulatory properties of the PFC layer, in order to successfully perform this task. The AG mechanism strengthens the PFC's intrinsic maintenance currents when the network is performing well, allowing the PFC to actively maintain currently relevant information (e.g., pay attention to the color dimension), and this sustained pattern of neural firing modulates processing pathways elsewhere in the model so as to amplify relevant information about the current stimulus. In a sense, the actively maintained patterns of PFC neural firing form a *working memory* trace of the current sorting rule. When the correct rule is changed (i.e., after 10 consecutive correct sorts), this actively maintained rule representation becomes invalid. If the network continues to actively maintain the invalid rule, allowing it to influence subsequent processing, perseverative errors will result. The AG mechanism prevents this by sending a DA based updating signal (*δ*(*t*) < 0) to the PFC when reward is expected but not delivered. This updating signal destabilizes the current PFC contents, allowing a new rule representation to be sampled, based on the current inputs to the PFC.

### Modeling Stroop

In order to simulate the relative prepotency of a stimulus dimension in the XT model, we manipulated the frequency with which one dimension was experienced as relevant during the initial developmental training received by the network. This target dimension, representing font color, was relevant over the course of developmental training only 25% as often as the other stimulus dimensions, such as word reading. A competition between the color naming and word reading pathways was simulated by co-activating features in this weaker dimension, corresponding to the color naming pathway, and a stronger dimension, representing the word reading pathway. In the XT model, the PFC layer provides the crucial top-down activation-based support, consistent with the model of [16], to resolve the competition in a manner appropriate for the current task. The time needed for output firing rate levels to stabilize (i.e., the *settling time*) in the face of this competition was used as an analog to human reaction time. Network settling time, measured as a number of simulated “time steps”, was scaled to milliseconds using a multiplicative scaling parameter selected so as to fit mean human data *only in the congruent word reading condition*. This scaling factor was then used, without modification, to translate model settling times in all conditions into millisecond measures that could be directly compared to human reaction times.

## Results

### Initial WCST Results

**Fig 2:**
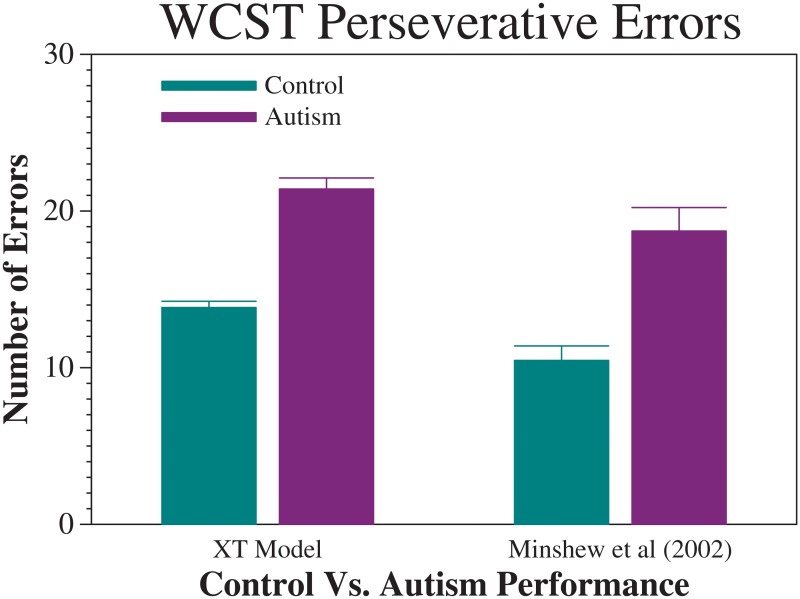
WCST Performance. XT Model & Human Data. Error bars represent standard error of the mean.

Our simulations of both healthy individuals (i.e., models with normal DA modulation) and persons with autism (i.e., models with reduced DA modulation) displayed a reliable difference between these groups, with the ASD models showing a reduction of cognitive flexibility as measured by an increase in perseverative errors. (See Table 1 for mean error results.) In particular, the number of perseverative errors was significantly higher in networks with reduced DA modulation, mirroring the behavior of people with ASD on this task, as reported in the literature [2,44–46]. Indeed, without performing any additional model fitting, the mean number of perseverative errors produced by the models provides a reasonable match to the human data of [46], as shown in Fig 2.

**Table 1:** WCST Model Results.

WCST Measure	Healthy	Autism	*F*(1,198)	*p* <
Total Errors	34.66	44.95	15.33	0.001
% Total Errors	35.80%	41.75%	13.23	0.001
Total Perseverative Errors	13.84	21.40	88.13	0.001
% Perseverative Errors	15.07%	20.38%	63.47	0.001

### Initial Stroop Results

**Fig 3:**
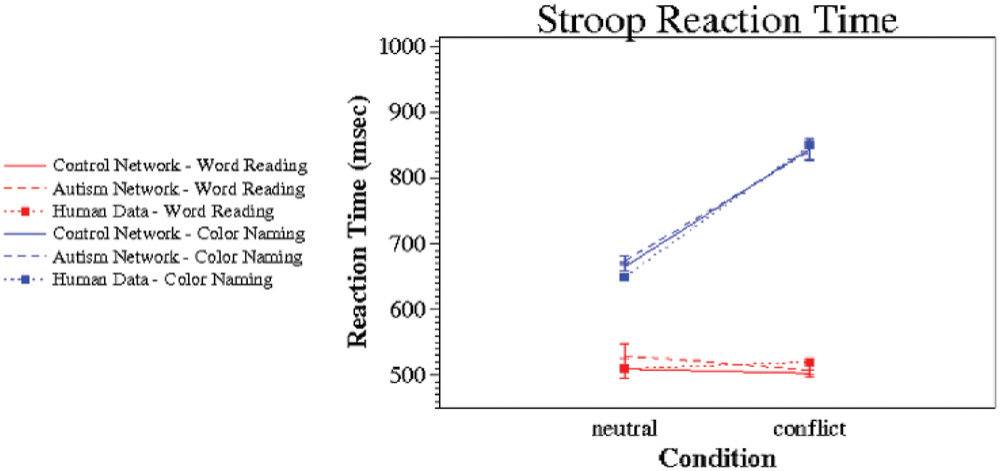
Stroop Performance. Healthy XT Model, Autistic XT Model, & Healthy Human Data. Error bars represent standard error of the mean.

Model performance on the Stroop task provided good quantitative and qualitative fits to human performance. (See Fig 3 for a comparison of reaction time performance for the healthy networks, the networks simulating people with autism, and healthy human data from [47].) The model with normal DA function displayed the classic Stroop reaction time results. The prepotent word reading dimension showed uniform reaction times across both congruent and conflict conditions, while the weaker color naming dimension exhibited a slowing in reaction times when the stimuli were incongruent, successfully demonstrating the Stroop effect. Importantly, the performance of the autistic models was virtually identical to the that of the healthy models, with no significant increase in the overall Stroop interference (*F*(1,198) = 0.62; *p* > 0.43). This is consistent with previously reported findings that people with autism show no reliable increase in Stroop interference, as compared with controls [2,11–13].

## Developmental Results

### Developmental Simulations

Our initial simulations involved the introduction of a DA deficit only after the model had undergone a standard process of simulated development. Thus, these initial simulations ignored the possibility that an early manifestation of a DA deficit might hinder the proper learning of PFC representations, introducing an impairment in cognitive control in the model that is not observed in people with autism. In order to address this shortcoming, a second set of simulations was conducted in which DA modulation was reduced in the autistic models from the very beginning of developmental training. In order to examine the developmental time course of cognitive control and cognitive flexibility in these models, Stroop and WCST performance were analyzed over the entire developmental period of the model (100 training epochs). Two groups of 10 networks, with each network treated as an individual for the purpose of analysis, were used. The autistic model group developed with reduced DA modulation (*κ* = 0.54), while the control model group underwent development training in the same manner as the original XT model (*κ* = 1.00).

At the end of developmental training, these groups of networks exhibited exactly the same pattern of results as seen in the initial simulations. In particular, no deficit appeared in the Stroop data, and perseverative errors remained elevated, as compared to controls, in WCST. Furthermore, the developmental time course data provided some insight into why executive deficits might appear late in autism, as described in the literature [18]. These developmental results are discussed next.

### PFC Representations

**Fig 4:**
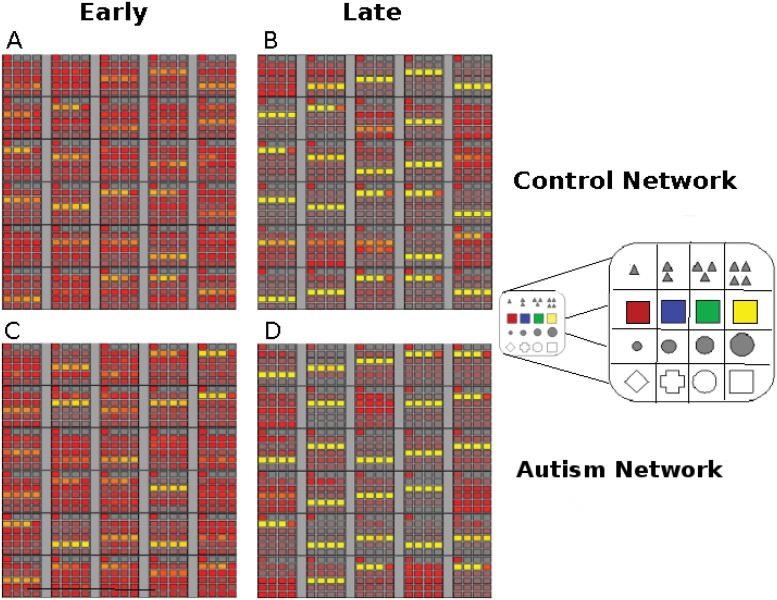
Comparing the development of PFC representations (synaptic strengths) between representative control (A, B) and autistic (C, D) networks. Comparisons are made early in development (Epoch 5, left side) and late (100 epochs, right side). Each image shows the strength of the PFC connections to the response layer, with the strength represented by the brightness of the smaller box (lighter means stronger). Each row designates connections to response units representing features in the same stimulus dimension (as illustrated in E). Images A and B show strength of the PFC representations in early and late stages respectively, and images C and D show the same information for the network modeling autistic performance. Note the lack of strong dimensional representations for both experimental groups early in development (A and C) and the relatively strong dimensional representations late (B and D). Please see text for a more in depth explanation.

Fig 4 plots the synaptic strengths from the PFC layer to the Response layer in the XT model. The top panels show synaptic weights for a representative healthy control network, and the bottom panels show the corresponding data for a representative autistic network. Each large rectangle in a panel corresponds to a modeled PFC unit, and each encapsulated small box corresponds to a modeled Response unit, with the strength of the connection from the given PFC unit to the given Response unit being reflected in the brightness of the box (i.e., lighter means stronger). Note that each row of small boxes designates connections to Response layer units representing features in the same stimulus dimension. Specifically, the presence of bright horizontal bands in these plots indicate that individual PFC units have learned, over the course of development, to modulate all features associated with a single stimulus dimension and none of the features associated with other dimensions. Thus, these plots can be used to examine the degree to which the PFC layer developed synaptic weights that encoded individual stimulus dimensions in the firing rates of individual PFC units as demonstrated in previous work [19]. The images on the hand side of Fig 4 (A and C) were generated early in the course of developmental training. At this point in development, both the autistic network and the control network lack strong dimensional weights. However, in Fig 4B and Fig 4D, which were generated at the end of developmental training, both networks have developed strong dimensional representations in PFC, as evidenced by the extremely salient horizontal bands of “strong” synaptic weights. Thus, appropriate PFC representations were acquired only slowly over the course of developmental training, and the DA manipulation used to model autism did not hinder the formation of these representations. Please note the following analysis is intended to provide the user with a intuition and qualitative understanding about the pattern and strength of connectivity that the network learned after development.

### Executive Dysfunction Development

**Fig 5:**
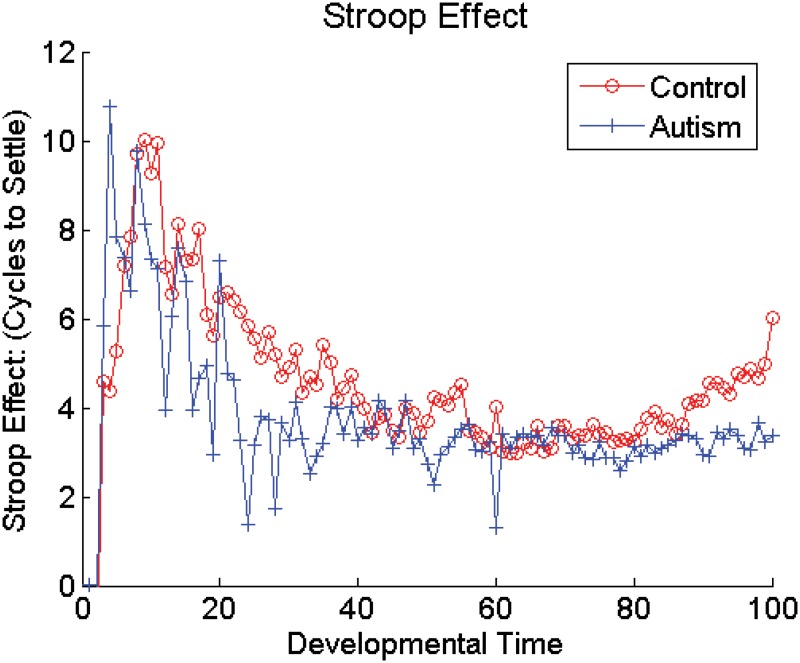
Stroop Interference in Models During Developmental Training.

**Fig 6:**
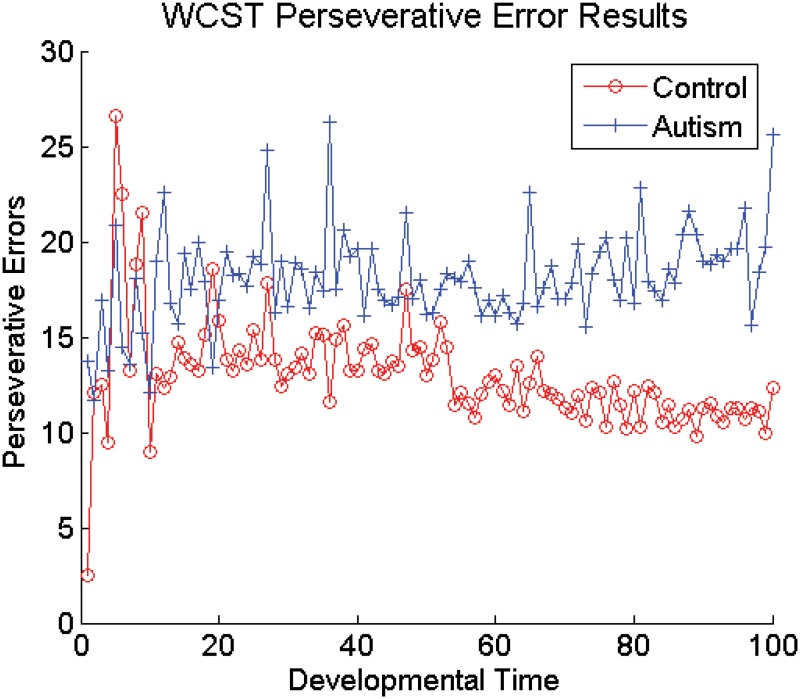
WCST Perseverative Errors in Models During Developmental Training.

Introducing damage to the DA based adaptive gating mechanism from the beginning of development still allows the XT model to capture autistic performance on tasks requiring cognitive flexibility and control. Stroop interference over the course of developmental training, averaged over the networks, is shown in Fig 5. Note the lack of an effect of the DA manipulation across the developmental period. Statistically, Stroop interference was significantly greater for the autistic networks during only 1 epoch out of the 100 experienced during development (*p* < 0.003), demonstrating robust cognitive control throughout the entirety of development. In contrast, Fig 6 plots WCST perseverative errors over the course of developmental training, averaged over the networks. This graph clearly shows a significant increase in the number of perseverative errors made by the autistic networks over time, with a significant difference between the autistic networks and the control networks appearing as early as epoch 12. During the first 50 epochs there was a significant difference (*p* < 0.05 on a one-tailed t-test) between the groups of networks during only 28% of the epochs. However, later in developmental training, during epochs 51–100, there were significantly more perseverative errors in the autistic networks during 88% of the epochs. In other words, neither the healthy models nor the models of autism showed a distinct advantage during the earliest stages of development, despite the presence of a reduction in DA modulation in one group, but not the other. The relative lack of executive deficits early in the development of these models could be due to the fact that both groups of networks lacked strong PFC representations early in training. Without strongly dimensional PFC representations, the models were forced to rely more heavily on synaptic plasticity in the Hidden layers (posterior cortical areas) to perform the tasks. Since weakening the DA based adaptive gating mechanism had no direct effect on these non-frontal areas of the model, neither group of networks showed any advantage over the other during this early stage. Thus, our model predicts that the lack of executive dysfunction observed in young children with autism results from the protracted development period required by the PFC in both normally developing children and children with autism.

## Discussion

These results demonstrate that dopamine may play a broader role in the development of ASD than has been previously imagined, providing a novel explanation of how patterns of ED may emerge in autism via disturbed PFC/DA interactions. Given the XT account of the role of PFC in executive control, we have shown that a single manipulation — reducing the efficacy of the DA signal — is sufficient to capture the pattern of performance exhibited by people with autism on basic tests of cognitive flexibility (WCST) and cognitive control (Stroop). In WCST, our models of autistic performance commit significantly more errors and, importantly, more perseverative errors when compared to simulated performance of normally functioning controls. This pattern of errors indicates that by weakening the effect of dopamine on gating of contents within the PFC, the ability of the model to flexibly adapt its behavior as task contingencies change is greatly reduced. In the simulations of Stroop performance, on the other hand, there is no significant change in performance when comparing the models with a reduced DA signal to those with no DA deficit. The Stroop model results indicate that reducing DA modulation of cells in PFC does not appear to affect the ability of PFC to actively maintain representations which can be used to influence subsequent processing in more posterior pathways. However, the WCST results point to a strong effect on the adaptive gating mechanism of PFC, resulting in a reduced ability to switch contexts in an appropriate manner.

Furthermore, we have demonstrated that weakening the DA based adaptive gating mechanism in the XT model over the entire course of PFC development continues to reflect both the strengths and weaknesses of autistic performance, while also providing some insight into the late appearance of cognitive flexibility deficits in autism. Specifically, the absence of strong PFC representations early in development masks the problematic PFC/DA interactions. As the model continues to develop, however, and the PFC representations strengthen, the weakened DA based adaptive gating mechanism manifests itself behaviorally as impaired cognitive flexibility. In this way, this model offers a candidate explanation for the late development of ED in autism [18].

Autism is an extremely heterogeneous disorder. Behavior is often diverse and varied, even within a single study. While data concerning the neural basis of these cognitive differences is rapidly accumulating, much mystery remains concerning the relationship between biology and behavior in autism. Indeed, an examination of the current literature quickly reveals a multitude of studies implicating nearly any brain region as a source of behavioral changes in ASD. The combination of diverse behavior and an abundance of neurological differences results in a stout challenge for the progression of any specific theory in this field. This paper demonstrates how the tools of computational cognitive neuroscience may provide some assistance in this endeavor by rigorously characterizing the behavioral effects of differences in brain function. Computational modeling methods of this kind have been applied to autism spectrum disorders by only a few researchers, and these past efforts have often been relatively cursory, lacking direct comparisons to empirical data [48–53]. By their very nature, computational cognitive neuroscience models add a certain amount of precision and rigor to scientific inquiry compared with purely verbal accounts. Underlying assumptions are necessarily quantified and unmasked, affording more precise and testable hypothesis. Constraints on these assumptions need to be made to maximize the utility of such models in autism research. Specifically, such models need to be constrained by both bottom-up (neurobiological mechanisms) and top-down (observed behavior) considerations. When used within these constraints, models of this kind provide a kind of “conceptual bridge” linking various levels of data and theoretical perspectives under a unifying framework.

Given the ample choices of brain areas to investigate, many with intriguing correlations to behavior in autism, why champion a closer look at the role of DA in autism? In truth, at this point, there is no “smoking gun” marking DA as the true underlying cause of any particular behavioral deficit observed in autism. Indeed, there is evidence for numerous neurochemical differences in people with ASD [54]. However, DA does provide a unique opportunity to provide a bridge linking seemingly disparate and complex behavior demonstrated by people with ASD. Neurally, dopamine affects nearly all of the brain areas associated with autistic behavior, including the cerebellum, amygdala, PFC, parietal lobes, and the hippocampus [9,55–59]. Increased seizure rates, motor abnormalities, stereotyped and repetitive behaviors, executive dysfunction, abnormal gaits, problems learning to follow eye gaze, and attentional abnormalities are all key components of behavior in autism, and all are linked tightly to the midbrain dopamine system [1,36,37,39,41,42,60–62]. This broad range of associations is at least suggestive that the dopaminergic system may play a prominent role in the etiology of autism.

Whether ED is better viewed as a primary cause of ASD or a reflection of other cognitive and biological differences is still an open and interesting question. However, there does appear to be some consensus to viewing behavioral flexibility as suspect. Juxtaposed against this general impairment is a myriad of data suggesting that sustained attention to parts of objects or situations often appears unimpaired when compared with matched controls. It is not our intention to suggest that all behavior in people with autism can be explained by the theory of executive dysfunction we describe in this report. Rather, we are attempting to provide a biologically inspired account that has the potential to explain a generally perplexing pattern of behavior demonstrated by many people with autism across a variety of tasks.

An important aspect of our future work is to directly compare performance by people with autism on WCST and Stroop using a within subject design, comparing performance to our model predictions. In short, we expect a trade-off between Stroop performance (as measured by the overall interference effect) and perseverative errors on the WCST. The smaller the Stroop interference effect (intact cognitive control), the greater the perseveration as measured by the WCST (impaired flexibility).

We have argued that perturbed DA/PFC interactions may lead to inflexibility in the updating of the contents of the PFC in people with autism. It is the goal of future research to demonstrate that this same mechanism may be capable of linking together a diverse range of abnormal patterns of behavior observed in people with autism. The modeling results reported here help to demonstrate the utility of formal computational models for investigating the relationship between brain and behavior in people with ASD. Using simulations, constrained and informed by both biology and observed behavior, precise and testable predictions of underlying mechanisms can be made, providing a theoretical bridge between psychological and anatomical theories of autism.
